# Dynamic changes of activated partial thromboplastin time and correlation with mortality in patients with severe fever with thrombocytopenia syndrome: A retrospective cohort study

**DOI:** 10.1371/journal.pntd.0013106

**Published:** 2025-05-22

**Authors:** Huan Wang, Sisi Fang, Hua Wang, Xin Zheng

**Affiliations:** 1 Department of Infectious Diseases, Union Hospital, Tongji Medical College, Huazhong University of Science and Technology, Wuhan, China; 2 School of Mathematics and Physics, China University of Geosciences, Wuhan, China; Australian Red Cross Lifeblood, AUSTRALIA

## Abstract

**Background:**

Hemorrhagic manifestations are highly prevalent in severe fever with thrombocytopenia syndrome (SFTS) patients and are significantly associated with fatal outcomes. In this study, we investigated the dynamic changes of activated partial thromboplastin time (APTT) and their association with mortality in SFTS patients.

**Methods:**

We conducted a retrospective study analyzing clinical data from SFTS patients admitted to our hospital between April 2017 and June 2024. The dynamic changes of APTT and their association with clinical outcomes were analyzed.

**Results:**

A total of 788 SFTS patients were enrolled in this study, among whom 96 (12.18%) died during hospitalization. Multivariate logistic regression identified prolonged APTT as an independent predictor of mortality, along with older age, neurological symptoms, higher viral load, and elevated creatinine levels. Prolonged APTT was observed in 568(72.08%) patients upon admission and was associated with the development of neurological symptoms, bleeding, intensive care unit (ICU) transfer, and mortality. APTT≥2.0 times the upper limit of normal (ULN) was associated with significantly higher mortality (55%) and an increased likelihood of ICU transfer (10%). Restricted cubic splines (RCS) analysis revealed that when the APTT level exceeded specific thresholds (49.86s upon admission and 53.61s at the peak during hospitalization), the predicted mortality of patients with SFTS increased with rising APTT levels. Kinetic analysis showed that APTT levels exhibited a declining trend during hospitalization and returned to the normal range by the 6th day in the survival group, while it gradually increased, reaching its peak on the 3rd day and then gradually decreased in the non-survival group.

**Conclusion:**

Prolonged APTT was prevalent among SFTS patients and was significantly associated with higher mortality. Monitoring APTT upon admission and its dynamic changes during hospitalization is recommended to enhance the management of SFTS patients.

## Author summary

Severe fever with thrombocytopenia syndrome is an emerging infectious disease with a high mortality rate. Hemorrhagic manifestations are highly prevalent in SFTS patients and are significantly associated with fatal outcomes. It is well established that abnormal coagulation function is closely linked to hemorrhagic manifestations. Understanding the intricate mechanisms by which coagulation dysfunction contributes to viral bleeding is crucial for developing targeted therapeutic strategies. This study provides a comprehensive analysis of serum coagulation parameters and investigates the dynamic changes of APTT and their association with mortality in SFTS patients. Our study demonstrate that SFTS patients exhibit significant coagulation dysregulation. Furthermore, univariate and multivariate logistic regression analyses identified prolonged APTT as an independent predictor of mortality. Kinetic analysis revealed that APTT levels in the survival group showed a declining trend during hospitalization and returned to the normal range by the 6th day, while it gradually increased, reaching its peak on the 3rd day and then gradually decreased in the non-survival group. In conclusion, this study highlights the critical role of coagulation dysregulation in SFTS, paving the way for further research in this field.

## 1. Introduction

Severe fever with thrombocytopenia syndrome is an emerging infectious disease caused by the SFTS virus (SFTSV), a member of the Bunyaviridae family. It was first reported in China in 2009 [1] and has since been identified across 27 provinces, 154 prefecture-level cities, and 533 counties in China [2]. The disease has rapidly spread to other regions, including South Korea, Japan, the United States, Vietnam and Myanmar [3–7]. It is characterized by high fever, thrombocytopenia, leukopenia, and multiple organ dysfunction, with a mortality rate of up to 7.8% [2]. Due to its high mortality rate and potential to cause global pandemic, SFTS was designated as one of the priority infectious diseases by the World Health Organization (WHO) in 2018 [8].

One of the notable complications associated with SFTS is the occurrence of hemorrhagic manifestations. Haemorrhagic signs has been observed in 35% of SFTS patients and are significantly associated with fatal outcomes [9]. The hemorrhagic manifestations are likely attributed to a combination of factors, including direct viral damage to endothelial cells, coagulation disorders, and platelet dysfunction [10]. Coagulation disorders are frequently observed in SFTS patients, with prolonged APTT being a notable significant of these disturbances [9]. Recent studies have emphasized the correlation between prolonged APTT and both the severity and poor prognosis of SFTS. Multivariate logistic regression analyses from previous studies have identified prolonged APTT as an independent predictor of mortality [11,12]. However, these studies did not account for viral load and only reported the APTT upon admission, without considering the dynamic change of APTT during the course of hospitalization.

In this study, we aimed to investigate the dynamic changes of APTT during hospitalization and their association with mortality in patients with SFTS. Thus, we sought to provide an easily accessible clinical indicator to assist clinicians in monitoring critically ill patients and facilitating timely intervention.

## 2. Patients and methods

### 2.1. Ethics statement

The clinical study was reviewed and successfully approved by the institutional review board of Tongji Medical College (2023-S093). The study was conducted in accordance with the principles of the Helsinki declaration of 1975, as revised in 1983.The requirement for getting written informed consent was waived due to the retrospective nature of the study.

### 2.2. Patients and data collection

A total of 816 laboratory-confirmed SFTS patients admitted to the Department of Infectious Disease at Wuhan Union Hospital between April 2017 and June 2024 were retrospectively analyzed. Laboratory-confirmed SFTS were meeting one or more of the following criteria: (a) detection of SFTSV RNA in the patient’s serum; (b) detection of IgM against SFTSV or a four-fold increase of serum IgG against SFTSV during the convalescent phase; (c) isolation of SFTSV through virus isolation techniques. After excluding 28 patients with no record of coagulation parameters, 788 SFTS patients eventually were ultimately enrolled in this study (S1 Fig). Upon admission, patients were stratified into four groups according to their APTT levels relative to the ULN: G1with APTT ≤1.0 times the ULN, G2 with APTT between 1.0 and 1.5 times the ULN, G3 with APTT between 1.5 and 2.0 times the ULN, G4 with APTT ≥2.0 times the ULN. Information of the patients’ demographic information, underlying diseases, clinical characteristics, laboratory findings, comorbidity and overall prognosis were collected from medical records by two trained physicians.

### 2.3. Statistical analysis

All statistical analyses were performed using R software (version 4.4.0; R Foundation for Statistical Computing, Vienna, Austria). Continuous variables were expressed as median (interquartile range) or mean (±standard deviation), and significant differences between the two groups were assessed using Student’s t-test or the nonparametric two-tailed MannWhitney U test. Categorical variables were presented as percentages(%), and significant differences between the two groups were assessed using the chi-square or Fisher’s exact test. Differences among multiple groups were determined using one-way analysis of variance (ANOVA) or the Kruskal-Wallis test. Multivariate logistic regression analyses were conducted to identify independent risk factor for mortality and prolonged APTT. Survival analysis according to the APTT levels across four groups was performed using Kaplan-Meier (KM) curves, and comparisons were made using the log-rank test. RCS were used to to further explore the association between APTT and SFTS mortality. The dynamic changes of APTT from admission to discharge or death were visualized using locally weighted scatterplot smoothing (LOESS). All statistical tests were two-tailed, and a p-value <0.05 was considered statistically signifcant.

## 3. Results

### 3.1. Baseline characteristics and potential independent risk factors for mortality of SFTS

Patients baseline demographic characteristics, underlying conditions, clinical manifestations, laboratory parameters upon admission and outcomes between survival and non-survival groups were summarized in Table 1. A total of 788 laboratory-confirmed SFTS patients were enrolled in this study, of whom 96 (12.18%) died during hospitalization. The median age was 63 (55–70) years old, and 332 (42.13%) patients were male. Regarding underlying conditions, 189 (23.98%) patients had hypertension, 134 (17.01%) had diabetes, 30 (3.81%) had chronic obstructive pulmonary disease (COPD), and 79 (10.03%) had chronic hepatitis B (CHB). Upon admission, 212 (26.90%) patients exhibited neurological symptoms, and 100 (12.69%) patients presented with hemorrhagic signs. The median length of hospitalization was 9 (7–12) days and 21 (2.66%) patients were transferred to ICU. The median APTT was 49.8s (42.38-60.53). The patients were divided into survival (n = 692) and non-survival group (n = 96). In the comparison between the two groups, the median age of patients in the non-survival group was significantly higher than that in the survival group (70 vs. 62, p < 0.001). Similarly, the prevalence of hypertension (40.62% vs. 21.68%, p < 0.001), neurological symptoms (68.75% vs. 21.1%, p < 0.001), hemorrhagic signs (21.88% vs. 11.42%, p = 0.0003), and need for ICU transfer (9.38% vs. 1.73%, p = 0.0039) were significantly higher in the non-survival group. Additionally, the non-survival group had significantly higher viral load_(Log10)_(5 vs. 2.85, p < 0.001), alanine aminotransferase (ALT) (116.5 vs. 76, p = 0.0002), aspartate aminotransferase (AST) (403 vs. 173, p < 0.001), globulin (25.8 vs. 25.2, p = 0.013), creatinine (91.35 vs. 67.4, p < 0.001), blood urea nitrogen (BUN) (7.69 vs. 4.63, p < 0.001), lactate dehydrogenase (LDH) (1446.5 vs. 717.5, p < 0.001), APTT (68.6 vs. 48.65, p < 0.001), D-dimer (5.18 vs. 2.31, p < 0.001), international normalized ratio (INR) (1.08 vs. 1.01, p = 0.0002), prothrombin time (PT) (13.7 vs. 13, p < 0.001), and thrombin time (TT) (42.35 vs. 23.45, p < 0.001). In contrast, the non-survival group had significantly shorter hospital stays (3 vs. 10, p < 0.001), lower platelet (PLT) counts (36 vs. 51, p < 0.001), lower lymphocyte counts (0.55 vs. 0.7, p < 0.001), lower monocyte counts (0.1 vs. 0.17, p < 0.001), and lower albumin (29.85 vs. 32, p = 0.0016). No significant differences were observed between the two groups in terms of gender, diabetes, COPD, CHB, white blood cell (WBC) counts, red blood cell (RBC) counts, neutrophil, total bilirubin (TBIL), or total protein (TP). Multivariate logistic regression analysis (Table 1) identified prolonged APTT (odds ratio (OR)=1.031, 95% confidence interval (CI): 1.0120-1.0505, p = 0.001) as an independent predictor of mortality, along with older age (OR=1.0931), neurological symptoms (OR=4.8288), higher viral load (OR=2.3618), and elevated creatinine (OR=1.0114).

**Table1:** Baseline characteristics and potential independent risk factors for mortality of SFTS.

Characteristic	Total(n = 788)	Survivor(n = 692)	Nonsurvivor(n = 96)	*p*
Age (yr)	63(55–70)	62(54–69)	70(63–73)	<0.001
Sex,male	332(42.13%)	292(42.20%)	40(41.67%)	0.922
Neurological symptoms	212(26.90%)	146(21.1%)	66(68.75%)	<0.001
Bleeding	100(12.69%)	79(11.42%)	21(21.88%)	0.004
Hypertension	189(23.98%)	150(21.68%)	39(40.62%)	<0.001
Diabetes	134(17.01%)	120(17.34%)	14(14.58%)	0.5
COPD	30(3.81%)	25(3.61%)	5(5.21%)	0.631
CHB	79(10.03%)	74(10.69%)	5(5.21%)	0.094
ICU transfer	21(2.66%)	12(1.73%)	9(9.38%)	<0.001
Hospital stay (day)	9 (7–12)	10 (8–12)	3(2–4)	<0.001
Viral load (Log10)	3.01 (2.01–3.96)	2.85 (2.00–3.71)	5.00 (3.90–5.66)	<0.001
WBC (×10^9^/L)	2.84 (1.75–4.77)	2.88 (1.77–4.86)	2.44 (1.69–4.24)	0.282
RBC (×10^12^/L)	4.21 (3.83–4.61)	4.21 (3.82–4.62)	4.22 (3.89–4.58)	0.692
Hb (g/L)	126 (115–139)	126 (115–139)	127 (116–139)	0.525
PLT (×10^9^/L)	50 (34–64)	51 (36–66)	36 (24–50)	<0.001
Lymphocyte (×10^9^/L)	0.68 (0.43–1.18)	0.7 (0.45–1.20)	0.55 (0.34–0.99)	0.005
Monocyte (×10^9^/L)	0.16 (0.08–0.37)	0.17 (0.08–0.38)	0.1 (0.06–0.22)	<0.001
Neutrophil (×10^9^/L)	1.6 (0.94–3.14)	1.61 (0.92–3.17)	1.53 (1.0975–2.77)	0.822
ALT (U/L)	79 (48–139.25)	76 (47–127.25)	116.5 (62.75–198.75)	<0.001
AST (U/L)	186 (100.75–362)	173 (94–321)	403 (222.25–705.75)	<0.001
TBIL (umol/L)	9.35 (6.9–12.7)	9.3 (6.9–12.63)	9.85 (7.18–13.05)	0.21
TP (g/L)	57.4 (53.3–61.3)	57.45 (53.4–61.33)	56.95 (52.15–60.93)	0.436
Albumin (g/L)	31.76 ± 4.43	31.95 ± 4.39	30.43 ± 4.49	0.002
Globulin (g/L)	25.25 (22.8–28.23)	25.2 (22.6–28.1)	25.8 (23.98–29.9)	0.013
Creatinine (umol/L)	69 (57.98–87.83)	67.4 (57.2–82.5)	91.35(69.7–152.55)	<0.001
BUN (mmol/L)	4.83 (3.46–6.6)	4.625 (3.35-6.23)	7.69 (5.53-11.34)	<0.001
LDH (U/L)	759.5 (487.75–1255.5)	717.5 (466–1116.25)	1446.5 (818–2291)	<0.001
APTT (s)	49.8 (42.38–60.53)	48.65 (41.6–57.63)	68.6 (56.25–85.75)	<0.001
D-dimer (ug/mL FEU)	2.55 (1.34–5.21)	2.31 (1.25–4.6)	5.18 (2.91–10.6)	<0.001
FIB (g/L)	2.48 (2.05–2.95)	2.5 (2.07–3.01)	2.32 (1.95–2.71)	0.018
INR	1.02 (0.94–1.13)	1.01 (0.94–1.11)	1.08 (1–1.21)	<0.001
PT (s)	13.1 (12.4–14.2)	13 (12.3–14.2)	13.7 (12.98–15.03)	<0.001
TT (s)	24 (20.28–31.53)	23.45 (19.98–28.9)	42.35 (26.78–65.88)	<0.001

Continuous variables were presented as median (interquartile range) or mean (±standard deviation), significant differences between the two groups were assessed using Student t test or the nonparametric two-tailed MannWhitney U test. Categorical variables were expressed as percentages(%), significant differences between the two groups were analyzed using the chi-square or Fisher exact test. p < 0.05 was considered statistically significant.

Abbreviations and normal range: SFTS, severe fever with thrombocytopenia syndrome; COPD, chronic obstructive pulmonary disease; CHB, chronic hepatitis B; ICU, intensive care unit; Viral load, < 100TCID50/ml; WBC, white blood cell, 3.5-9.5 × 10^9^/L; RBC, red blood cell, 3.8-5.1 × 10^12^/L; Hb, hemoglobin, 115-150g/L; PLT, platelet,125–350 × 10^9^/L; ALT, alanine aminotransferase, 5-35U/L; AST, aspartate aminotransferase, 8-40U/L; TBIL, total bilirubin, 5.1-19umol/L; TP, total protein, 64-83g/L; Albumin, 35-55g/L; Globulin, 20-30g/L; Creatinine, 44–106umol/L; BUN, blood urea nitrogen, 2.9-8.2mmol/L; LDH, lactate dehydrogenase, 109-245U/L; APTT, activated partial thromboplastin time, 28-43.5s; D-dimer, < 0.5ug/mL FEU; FEU, fibrinogen equivalent unit; FIB, fibrinogen, 2.0-4.0g/l; INR, international normalized ratio, 0.8-1.2; PT, prothrombin time, 11-14.2s; TT, thrombin time, 14-21s.

### 3.2. Association between prolonged APTT and clinical outcomes

Table 2 describes the baseline characteristics of SFTS patients categorized according to their APTT levels on admission. Prolonged APTT was observed in 72.08% patients upon admission and was associated with the development of neurological symptoms, bleeding, ICU transfer, and mortality. No significant differences were found in the prevalence of comorbidities such as hypertension, diabetes, COPD, and CHB among the four groups. Most patients were classified into G2 (395 cases), characterized by mild prolongation of APTT. G1 accounts for 220 cases, had the lowest median age (61 years) and the highest proportion of female patients(66.82%). This group exhibited the lowest rates of neurological complications(14.09%), bleeding(9.55%), mortality(1.82%), and ICU transfer(0%). Additionally, patients in G1 have the mildest clinical conditions, with the lowest levels of SFTSV viral load, liver function biomarkers (ALT, AST and Tbil), renal function biomarkers (creatinine and BUN), myocardial injury-related biomarkers (LDH) and coagulation function biomarkers (INR, PT and TT), as well as the highest level of PLT and FIB, all p < 0.001. In contrast, G3 included 133 cases and was characterized by the longest hospital stay (11 days), the oldest median age (66 years), the highest proportion of male patients (52.63%), and the highest incidence of diabetes (20.3%). G4 has a total of 40 cases, was associated with significantly higher mortality(55%) and an increased likelihood of ICU transfer(10%). This group had the highest levels of SFTSV viral load, liver function biomarkers (ALT, AST and Tbil), renal function biomarkers (creatinine and BUN), myocardial injury-related biomarkers (LDH) and coagulation function biomarkers (INR, PT and TT), as well as the lowest levels of PLT and FIB, all p < 0.001. Furthermore, a positive correlation was observed between APTT levels and SFTSV viral load, ALT, AST, and LDH, while, a negative correlation was found between APTT levels and PLT counts upon admission.

**Table 2:** Characteristics and outcomes of participants categorized by serum APTT on admission.

		baseline APTT				*P* value
Characteristic	Total (n = 788)	G1 ≤ 1.0ULN (n = 220)	G2 1.0-1.5ULN (n = 395)	G3 1.5-2.0 ULN (n = 133)	G4 ≥ 2.0 ULN (n = 40)	
Age (yr)	63(55–70)	61(54–68)	63(55–70)	66(57–71)	63(55.75–70)	0.0331
Sex, male	332(42.13%)	73(33.18%)	171(43.29%)	70(52.63%)	18(45%)	0.0035
**Comorbidity**						
Hypertention	189(23.98%)	50(22.73%)	89(22.53%)	38(28.57%)	12(30%)	0.395
Diabetes	134(17.01%)	31(14.09%)	73(18.48%)	27(20.30%)	3(7.50%)	0.138
COPD	30(3.81%)	6(2.73%)	14(3.54%)	7(5.26%)	3(7.50%)	0.309
CHB	79(10.03%)	21(9.55%)	41(10.38%)	14(10.53%)	3(7.50%)	0.967
**Complications**						
Neurological symptoms	212(26.90%)	31(14.09%)	104(26.33%)	55(41.35%)	22(55.00%)	<0.001
Bleeding	100(12.69%)	21(9.55%)	44(11.14%)	23(17.29%)	12(30.00%)	0.001
**Laboratory parameters**						
Viral load (Log10)	3.01(2.01–3.96)	2.00(0.96–2.83)	3.07(2.34–3.80)	4.05(3.16–5.00)	5.07(4.25–5.93)	<0.001
WBC (×10^9^/L)	2.84(1.75–4.77)	3.62(2.33–6.19)	2.39(1.58–4.15)	2.85(1.62–4.01)	2.86(1.72–4.71)	<0.001
RBC (×10^12^/L)	4.21(3.83–4.61)	4.08(3.74–4.45)	4.23(3.87–4.64)	4.34(3.92–4.81)	4.20(3.97–4.46)	<0.001
Hb (g/L)	126(115–139)	123(111.75–134.25)	128(116–140)	128(116–140)	124(118.75–134.50)	<0.001
PLT (×10^9^/L)	50(34–64)	61.50(42–81.25)	50(36–61)	38(27–49)	31.50(21.75–53.75)	<0.001
Lymphocyte (×10^9^/L)	0.68(0.43–1.18)	1.09(0.72–1.58)	0.55(0.38–0.90)	0.60(0.41–0.98)	0.75(0.44–1.39)	<0.001
Monocyte (×10^9^/L)	0.16(0.08–0.37)	0.34(0.1775–0.54)	0.13(0.07–0.265)	0.12(0.06–0.22)	0.165(0.0675–0.3025)	<0.001
Neutrophil (×10^9^/L)	1.60(0.94–3.14)	1.92(1.02–4.13)	1.5(0.88–3.01)	1.55(1.07–2.94)	1.80(0.82–2.71)	0.054
ALT (U/L)	79(48–139.25)	64(44–120.50)	72(44.50–115.50)	119(79–186)	168(81–302.75)	<0.001
AST (U/L)	186(100.75–362)	112.50(70–195)	178(106–302)	407(258–634)	706(320.50–1057.75)	<0.001
TBIL (umol/L)	9.35(6.90–12.70)	10.70(7.78–15.20)	8.40(6.60–10.95)	9.30(6.80–12.40)	12.30(10.05–17.53)	<0.001
TP (g/L)	57.44 ± 6.10	58.84 ± 6.48	57.72 ± 5.69	55.25 ± 5.97	54.20 ± 5.34	<0.001
Albumin (g/L)	31.76 ± 4.43	32.48 ± 4.63	32.34 ± 4.22	29.77 ± 4.00	28.73 ± 3.47	<0.001
Globulin (g/L)	25.25(22.8–28.23)	25.60(23.40–29.13)	25.20(22.60–28.15)	25.20(22.80–27.80)	24.75(22.15–29.43)	0.19
Creatinine (umol/L)	69(57.98–87.83)	60.10(51.25–70.05)	71.40(60–89.70)	77.70(63.50–106.50)	73.25(64.40–110.08)	<0.001
BUN (mmol/L)	4.83(3.46-6.60)	4.18(3.29-5.42)	5.02(3.47-6.70)	5.40(4.30-7.90)	5.83(4.19-8.86)	<0.001
LDH (U/L)	759.50(487.75-1255.50)	532.50(382.25-797.75)	725(496-1021.50)	1419(913-1893)	2088.50(1496.25-2898.75)	<0.001
D-dimer (ug/mL FEU)	2.55(1.34–5.21)	1.24(0.78–1.90)	2.57(1.61–4.77)	5.45(3.50–10.59)	9.87(5.52–18.19)	<0.001
FIB (g/L)	2.48(2.05–2.95)	2.85(2.05–3.46)	2.51(2.22–2.88)	2.20(1.70–2.54)	2.01(1.73–2.32)	<0.001
INR	1.02(0.94–1.13)	0.99(0.92–1.16)	1.01(0.94–1.08)	1.08(0.99–1.20)	1.18(1.05–1.31)	<0.001
PT (s)	13.10(12.40–14.20)	12.80(12.10–14.43)	13(12.35–13.80)	13.70(12.90–14.60)	14.70(13.60–16.28)	<0.001
TT (s)	24(20.28–31.53)	20.75(16.40–23.50)	24.10(21.05–28.45)	38.90(27.70–59.40)	73.60(59.95–105.63)	<0.001
**Outcomes**						
Mortality	96(12.18%)	4(1.82%)	32(8.1%)	38(28.57%)	22(55%)	<0.001
ICU transfer	21(2.66%)	0(0%)	8(2.03%)	9(6.77%)	4(10%)	<0.001
Hospital stay (day)	9(7-12)	8(6-10)	10(8-12.5)	11(5-13)	5(2-13.25)	<0.001

Differences among multiple groups were determined using one-way analysis of variance or the Kruskal-Wallis test. p < 0.05 was considered statistically significant.

G1, group1 with APTT≤1.0 ULN; G2, group2 with APTT from 1 to 1.5ULN; G3, group3 with APTT from 1.5 to 2.0 ULN; G4, group4 with APTT ≥2ULN, normal range of APTT, 28s-43.5s.

Abbreviations: ULN, upper limit of normal

**Fig 1:**
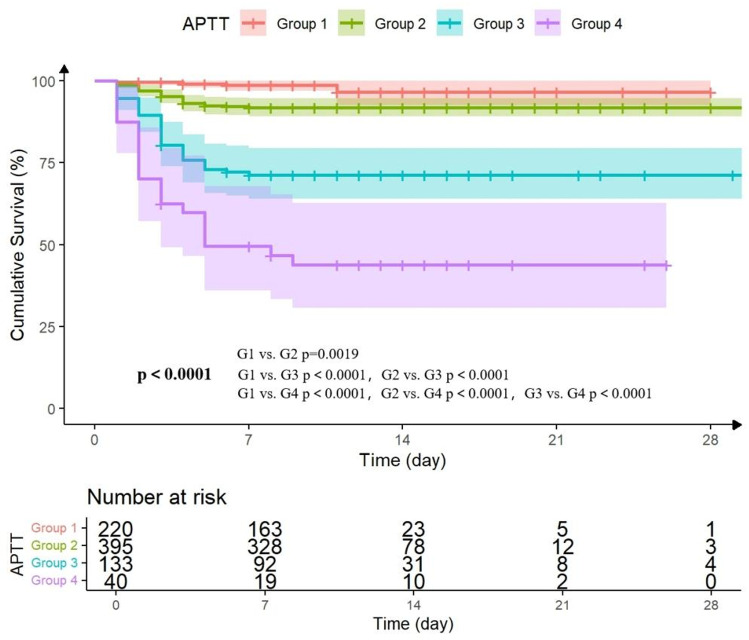
Kaplan-Meier survival analysis curves according to the APTT levels between four groups. Further analysis was performed to evaluate the risk stratification value of APTT measured upon admission and at peak of hospitalization for 28-day mortality across various subgroups. These subgroups included age, gender, neurological symptoms, hemorrhagic signs, hypertension and diabetes (S2 Fig). Subgroup forest plot analysis revealed that the predictive power of APTT was not influenced by age, gender, comorbidity and complications, demonstrating its robust predictive ability.

The results of the Kaplan–Meier survival analysis are presented in Fig 1, showing significant differences in survival probabilities among the four groups (G1 vs. G2p=0.0019, G1 vs. G3p< 0.0001, G1 vs. G4 p< 0.0001, G2 vs. G3p< 0.0001, G2 vs. G4p< 0.0001, G3 vs. G4p < 0.001). Prolonged APTT was significantly associated with an increased risk of 28-day all-cause mortality (p < 0.0001). The highest survival rate was observed in G1, while the lowest survival rate was noted in G4.

**Fig 2:**
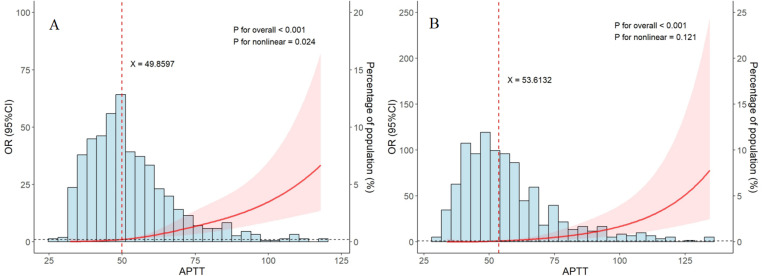
Restricted cubic spline models depicting the relationship between APTT and mortality risk in SFTS. A, APTT upon admission; B, APTT peak value of hospitalization. The relative frequency of APTT is represnted by the blue bars. The 95% CI of the adjusted odds ratio (OR) is represented by the red-shaded areas. Abbreviations: CI, confidence interval; OR, odds ratios;.

The association between APTT (upon admission or peak of hospitalization) and 28-day mortality of SFTS was assessed on a continuous scale using RCS curves based on a logistic regression model. As depicted in Fig 2A, APTT values were predominantly distributed around 50s. A J-shaped correlation was exhibited between APTT upon admission and 28-day mortality. The risk of mortality remained relatively stable until the predicted APTT upon admission reached approximately 49.86 seconds, after which it increased rapidly (p for overall < 0.001). Similarly, Fig 2B illustrates the relationship between peak APTT during hospitalization and mortality. The risk of mortality remained relatively constant until the predicted peak APTT reached around 53.61s, after which it rose sharply (p for overall < 0.001). Additionally, a significant nonlinear relationship was observed between APTT upon admission and mortality (p for nonlinearity = 0.024).

### 3.3. Dynamic change of APTT in survivor and non-survivor groups

**Fig 3:**
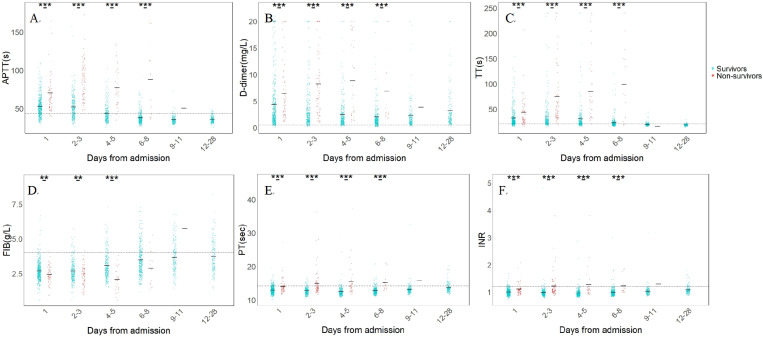
Kinetics of coagulation parameters between the survivors and non-survivors during hospitalization. All parameters were analyzed at different time intervals for the entire hospital stay. The black solid line represents median of reference parameters, The ULN of reference parameters were depicted with a black dotted line. Three asterisk (***) indicates p < 0.001, two asterisks (**) indicate p < 0.01, one asterisks (*) indicate p < 0.05.

**Fig 4:**
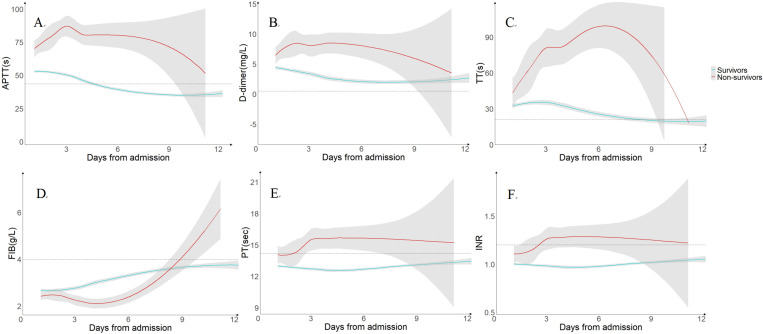
Dynamic profile of coagulation parameters between survivors and non-survivors in patients with SFTS.

We conducted a kinetics analysis of coagulation parameters, tracking their changes from day 1 to day 28 or until discharge (Fig 3). The trajectories of coagulation parameters from day 1 to day 12 or discharge are presented in Fig 4. At each time point during hospitalization, the non-survivors exhibited significantly higher levels of APTT, D-dimer, TT, PT, and INR compared to survivors (p < 0.001). In contrast, fibrinogen (FIB) levels were significantly lower in non-survivors than in survivors from day 1 to day 5 (p < 0.001), but gradually increased thereafter, surpassing survivor levels by Day 9.. Among survivors, APTT, D-dimer and TT levels peaked at admission, followed by a declining trend. APTT and TT returned to normal range by day 6 and day 9, respectively, whereas D-dimer remained elevated above the normal upper limit until discharged. In non-survivors, APTT, D-dimer, and TT progressively increased, peaking on day 3, day 5, and day 8, respectively, and then gradually decreased.

### 3.4. Potential factors associated with prolonged APTT in patients with SFTS

Building upon previous results (Table 2, p < 0.05), we further explored potential risk factors associated with prolonged APTT (Table 3). Multivariate regression analysis revealed that higher viral load (OR=1.5019, p < 0.001), elevated AST (OR=1.0061, p < 0.001), lower lymphocyte (OR=0.5676, p = 0.006) and TBIL (OR=0.0.9216, p < 0.001) were potential risk factors for prolonged APTT≥1.0 ULN in patients with SFTS. For more severe APTT prolongation (≥2.0 × ULN), higher viral load (OR=2.4015, p < 0.001), elevated AST (OR=1.0011, p = 0.003), along with lymphocyte count(OR=2.0016, p = 0.032) were potential risk factors

**Table 3:** Multivariate logistic regression analysis on the risk factors associated with prolonged APTT.

	APTT≥1.0ULN		APTT≥2.0ULN	
**Factors**	OR(95%CI)	p	OR(95%CI)	p
Viralload (Log10)	1.5019 (1.2413 ~ 1.8173)	<0.001	2.4015 (1.8167 - 3.1744)	<0.001
Lymphocyte (×10^9^/L)	0.5676 (0.3788 ~ 0.8504)	0.006	2.0016 (1.0627 - 3.7701)	0.032
AST (U/L)	1.0061 (1.0033 ~ 1.0089)	<0.001	1.0011 (1.0004 - 1.0018)	0.003
TBIL (umol/L)	0.9216 (0.8914 ~ 0.9528)	<0.001	1.0293 (0.9904 - 1.0698)	0.142

## 4. Discussion

We present a large-scale retrospective population-based study to explore the dynamic change of coagulation parameters, espencially APTT, and its association with mortality in patients with SFTS. Our findings revealed a high prevalence of prolonged APTT among SFTS patients. Multivariate analyses identified APTT, along with age, neurological symptoms, viral load, and creatinine, as independent predictors of increased mortality risk. Notably, patients with higher APTT demonstrate lower survival probability. The longitudinal analysis revealed distinct trajectories of coagulation parameters between survivors and non-survivors, APTT showed a declining trend during hospitalization and returned to the normal range in the survival group, while it gradually increased, reaching its peak on the 3rd day and then gradually decreased in the non-survival group. Furthermore, viral load, lymphocyte, AST, and TBIL were significantly associated with prolonged APTT.

Our study identified older age, with neurological symptoms, high viral load, elevated levels of creatinine, and prolonged APTT as significant risk factors for mortality in SFTS patients. Notably, viral load and neurological symptoms emerged as the most prominent predictors, consistent with previous studies [9,12,13]. Prior large-sample retrospective studies [9,13] demonstrated that non-survivors were typically older and had higher viral loads, with a positive correlation between viral load and age. This association may reflect greater tick exposure among elderly rural populations engaged in agricultural activities, combined with limited healthcare access [9]. Furthermore, age-related comorbidities, espencially hypertention and diabetis likely contribute to immune dysfunction, increasing susceptibility to severe viral infections and hyperviremia [14]. The observed risk factors (neurological symptoms, elevated viral load, creatinine, and APTT) align with reported predictors of poor outcomes in both COVID-19 and severe SFTS infections [11,15-16]. Japanese autopsy studies of SFTS cases found extremely high SFTSV-RNA levels in the heart, liver, and kidney. Neurological symptoms and multi-organ damage may result from direct virus invasion or indirect mechanisms including cytokine storm and immune-mediated injury [17,18]. In elderly patients with comorbidities, pre-existing immune compromise facilitates rapid viral replication, leading to high-grade viremia. Subsequent viral dissemination and/or immune-mediated injury can induce multi-organ dysfunction involving the nervous system, myocardium, kidneys, and coagulation pathways, potentially progressing to life-threatening hemorrhage or multi-organ failure. Consequently, it is important for elderly individuals to take protective measures when engaging in outdoor activities. After being infected, Antiviral drugs such as favipiravir should be used as soon as possible [19,20], monitor organ functions closely, and enhance supportive treatment.

In our study, prolonged APTT was observed in 72.08% of SFTS patients upon admission. This proportion was lower than that reported by Tang et al. (82.1%) [21] in SFTS patients but higher than rates documented in dengue fever (42.91%) [22], and hemorrhagic fever with renal syndrome (HFRS; 53.96%) [23]. Notably, non-survivors exhibited significantly higher APTT levels at admission compared to survivors. The case fatality rate was highest (55%) among patients with APTT ≥2.0 ULN, whereas it markedly decreased to 1.82% in those with with APTT≤1.0 ULN. Additionally, D-dimer and TT were significantly elevated in the non-survival group. In contrast, PT (reflecting extrinsic coagulation pathway activity), INR, and FIB levels remained within the normal range, aligning with previous studies [21,24]. The typically normal PT observed in SFTS patients may be attributed to the neutralization of heparin-like substances present in commercial PT reagents by polybrene [25].

In our research, the average APTT at admission was 49s in the overall patient cohort, while non-survivors exhibited a significantly prolonged APTT (68.6s). These findings align with a previous study from Anhui (51.9 vs 65.4s in overall patients and non-survivors, respectively) [26], shorter than those reported by studies in Wuhan (67.58 vs 83s) [21,24], longer than the study from the north China (43.5 vs 55.7s) [12]. RCS analysis revealed a J-shaped association between APTT levels and 28-day mortality, with mortality rates increasing sharply when APTT exceeded 49.86s at admission (53.61s during hospitalization), consistent with the findings of Peng W et al. [12]. Notably, clinicians should be particularly vigilant for patients with APTT levels > 49.86s at admission (53.61s during hospitalization). Subgroup analysis further demonstrated that the prognostic value of APTT remained robust, unaffected by age, gender, comorbidities, or complications. Additionally, dynamic change analysis indicated that the APTT progressively declined in survivors but continued to rise in non-survivors during the three days preceding hospitalization, corroborating prior research [24,27]. This findings highlight the importance of monitoring coagulation parameters, particularly APTT, in the days leading up to hospitalization, for early risk stratification and clinical decision-making.

APTT is a fundamental test for evaluating of the intrinsic pathway. rolonged APTT may result from deficiencies in Factors XII, XI, IX & VIII, vitamin K deficiency, liver disease, disseminated intravascular coagulation (DIC), or the presence of lupus anticoagulants [28]. Similar to other viral hemorrhagic fevers, such as dengue fever [22], Crimean-Conga hemorrhagic fever [29], and HFRS [30], the mechanisms underlying APTT prolongation in SFTS patients may involve:(a) Endothelial glycosaminoglycans shedding, leading to increased endogenous heparin-like substances in the circulation [21]; (b) Factor XI deficiency [31]; (c) Direct viral-induced endothelial cell dysfunction, triggering coagulation activation and factor consumption [32]; (d) Impaired hepatic synthesis of coagulation factors due to acute liver injury. Our findings demonstrate that higher viral load and elevated AST were associated with an increased the risk of prolonged APTT (≥1.0 ULN), suggesting that viral infection and acute liver injury are key contributors to APTT prolongation in SFTS patients.

There were several limitations in our study. Firstly, this is a single-center retrospective study, future multicenter studies are necessary to validate our findings. Secondly, variables associated with mortality, such as treatment, was not included in the analysis. Furthermore, the exclusion of some patients due to the absence of viral load and coagulation parameters could potentially introduce selection bias into the study.

In conclusion, this study examined the dynamic changes of coagulation parameters in SFTS patients and identified APTT as a potential risk factor for mortality. Monitering APTT during initial hospitalization (particularly upon admission and throughout the first 72 hours) could provide clinically actionable prognostic information. These findings offer valuable insights for risk stratification and may guide timely clinical decision-making to improve outcomes in SFTS management.

## Supporting information

S1 Table Multivariate logistic regression analysis on the risk factors associated with mortality of SFTS. (DOCX)

S1 Fig Flowchart of participants enrollment in the study. (TIF)

S2 Fig Forest plots of APTT for 28-day mortality in different subgroups. A, APTT on admission; B, APTT peak value of hospitalization.
(TIF)

S1 Data Anonymized individual patient data underlying all analyses.
(XLSX)
