# Supplementary material for: Dynamic changes of activated partial thromboplastin time and correlation with mortality in patients with severe fever with thrombocytopenia syndrome: A retrospective cohort study
Source: PLoS Negl Trop Dis. 2025 May 22;19(5):e0013106. doi: 10.1371/journal.pntd.0013106 (PMC12140419; doi:10.1371/journal.pntd.0013106)
Supplement: S1 Table — (DOCX) [file pntd.0013106.s001.docx]

**S1 Table Multivariate logistic regression analysis on the risk factors associated with mortality of SFTS**

| Factors | Z | p | OR | 95% CI |
| --- | --- | --- | --- | --- |
| Age (yr) | 4.4439 | <0.001 | 1.0931 | (1.0510-1.1368) |
| Neurological symptoms | 4.9459 | <0.001 | 4.8288 | (2.5873-9.0122) |
| Viralload (Log10) | 5.981 | <0.001 | 2.3618 | (1.7821-3.1300) |
| Creatinine (umol/L) | 4.4534 | <0.001 | 1.0114 | (1.0064-1.0164) |
| LDH (U/L) | 1.7991 | 0.072 | 1.0004 | (1.0000-1.0008) |
| APTT (s) | 3.2071 | 0.001 | 1.031 | (1.0120-1.0505) |
| D-dimer (ug/mL FEU) | -1.4739 | 0.141 | 0.953 | (0.8940-1.0160) |
| Hypertension | 1.5357 | 0.125 | 1.6358 | (0.8729-3.0655) |
