# Supplementary figures and images for: Dynamic changes of activated partial thromboplastin time and correlation with mortality in patients with severe fever with thrombocytopenia syndrome: A retrospective cohort study
Source: PLoS Negl Trop Dis. 2025 May 22;19(5):e0013106. doi: 10.1371/journal.pntd.0013106 (PMC12140419; doi:10.1371/journal.pntd.0013106)

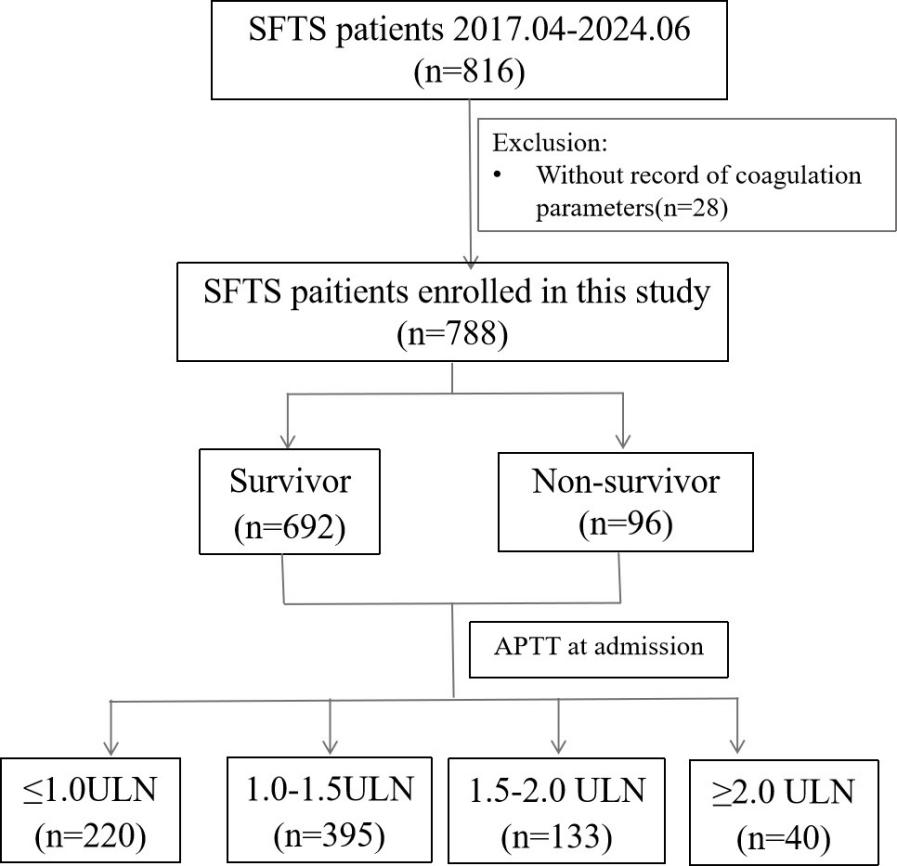

Supplement: S1 Fig — (TIF) [file pntd.0013106.s002.tif]

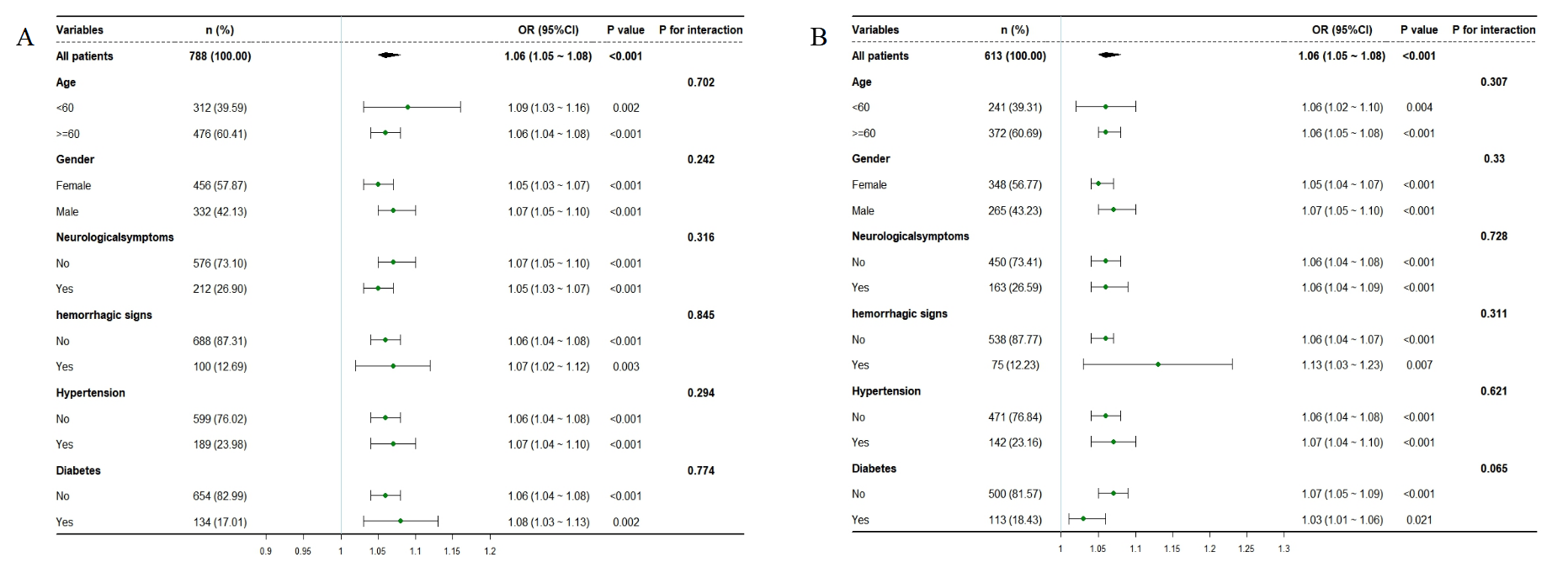

Supplement: S2 Fig — A, APTT on admission; B, APTT peak value of hospitalization. (TIF) [file pntd.0013106.s003.tif]
